# Role of the biomarkers for the diagnosis of Creutzfeldt-Jakob disease

**Published:** 2016

**Authors:** A Dulamea, E Solomon

**Affiliations:** * ”Carol Davila” University of Medicine and Pharmacy, Bucharest, Romania; ** Department of Neurology, Fundeni Clinical Institute, Bucharest, Romania

**Keywords:** Creutzfeldt-Jakob disease, 14-3-3 protein, magnetic resonance imaging, rapidly progressive dementia, periodic sharp wave complexes

## Abstract

**Objective: **Sporadic Creutzfeldt-Jakob disease (CJD) is a human prion disease, rapidly progressive and fatal, characterized by spongiform encephalopathy. The characteristic triad of signs - rapidly progressive dementia, myoclonus and periodic sharp wave complexes (PSWC) on electroencephalography (EEG) - usually appear in the late stages of the disease. The clinical diagnosis of CJD ante-mortem involves the exclusion of the rapidly progressive non-prionic dementias, the definitive diagnosis requiring brain tissue confirmation. Authors evaluated the methods of clinical diagnosis for sporadic CJD.

**Methods: **This study retrospectively reviewed the medical records of patients diagnosed with probable sporadic CJD, based on brain magnetic resonance imaging (MRI), EEG, cerebrospinal fluid (CSF) analysis and extensive laboratory work-up.

**Results:** Four patients with a mean age of 67 years were included in our study. The mean duration from diagnosis until death was of 3.2 weeks. The clinical features of the disease at onset were atypical. In the final stage of the disease, all patients presented rapidly progressive dementia and myoclonus. High levels of 14-3-3 protein and tau protein and normal levels of amyloid β1-42 were found at CSF analysis, in all patients. PSWC on EEG were present in 3 out of 4 patients at different moments of the disease. MRI showed hyperintense lesions in brain cortex, caudate nucleus, and putamen on T2, FLAIR, and DWI.

**Conclusion:** CJD may present various clinical features and, since brain biopsy is usually difficult to perform, a combination of biomarkers is useful in order to establish the diagnosis in the early phase of the disease.

## Introduction

The human prion diseases are a group of rare and fatal diseases caused by the misfolding of a normal prion protein (PrPc) into an abnormal conformation (the prion or PrPsc) which self-propagates in the absence of a nucleic acid [**1**,**2**]. PrPsc is mostly confined to the central nervous system. However, small quantities of PrPsc are present in many tissues and body fluids even at early pre-symptomatic stages of the disease [**3**]. In humans, prion diseases result from contamination, genetic inheritance, or sporadic events. The host susceptibility is influenced by the prion protein-encoding gene, PRNP. Sporadic Creutzfeldt-Jakob disease (sCJD) is the most common form of human prion disease, death usually occurring within a year or less from the onset [**4**,**5**], the disease usually affecting persons aged 50 to 70 years old [**6**]. The differential diagnosis includes neurodegenerative diseases (Alzheimer disease, Lewy body disease, and frontotemporal dementia), acute neurological diseases (e.g. CNS inflammation, tumor, and stroke), and non-neurological diseases (e.g. psychiatric, metabolic, toxic). A diagnosis of probable sporadic CJD is based on clinical signs of rapidly progressive dementia and at least two of the following clinical features: myoclonus, visual or cerebellar disturbances, pyramidal or extrapyramidal dysfunctions, akinetic mutism, evidence of paroxysmal sharp wave complexes (PSWC) as characteristic encephalographic (EEG) findings, positive 14-3-3 protein assay in the cerebrospinal fluid (CSF) [**7**] and a clinical evolution until death of less than two years [**8**]. Magnetic resonance imaging (MRI) findings such as high signal on diffusion-weighted images (DWI) and fluid attenuated inversion recovery (FLAIR) in at least two cortical regions (temporal, parietal or occipital) or both caudate nuclei and putamen are useful diagnostic tools especially in early stages of the disease [**9**]. The diagnosis of definite sporadic CJD involves neuropathological confirmation and/ or confirmation of protease-resistant prion protein and/ or the presence of scrapie-associated fibrils in CSF [**7**].

## Material and methods

The authors retrospectively reviewed the medical records, brain MRI images, EEG findings, and CSF analysis of patients diagnosed with probable sporadic Creutzfeldt-Jakob disease (CJD) between 2012 and 2014 in the Neurology Clinic of Fundeni Clinical Institute. The inclusion criteria were: (1) diagnosis of probable sCJD according to World Health Organization criteria [**9**] completed by MRI-CJD Consortium criteria for sporadic Creutzfeldt-Jakob disease [**8**], (2) exclusion of other causes of rapidly progressive dementia.

**Case 1**

**Fig. 1:**
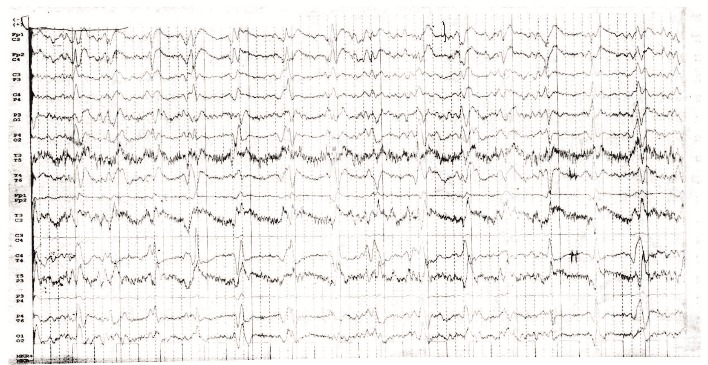
PSCW on the right temporal and parietal leads, with a mean frequency of 1.0 Hz and a mean voltage of 100 μV

A 72-year-old female had presented progressive onset of temporal and visuospatial disorientation, cognitive impairment, language disorder, and unsteady gait for two weeks. She had no previous medical history and denied any family history of dementia. The neurological examination revealed temporal and spatial disorientation, nystagmus, gait with small steps, paraparesis (4/ 5 BMRC), movement of all limbs to painful stimuli, brisk stretch reflexes with extensor plantar response, visual inattention on the left side and spatial neglect, conduction aphasia (deficit of verbal comprehension for complex orders, execution of simple commands with delay, dysnomia, disorder of repetition for words and phrases) and ideomotor apraxia. The neuropsychological assessment revealed temporal disorientation, attention deficit, anterograde hypomnesia, difficulties in executing complex orders, tendency towards perseveration and disexecutive syndrome especially on drawing tests. At the Mini-Mental State Examination (MMSE) the patient’s score was 17/ 30, at Clock-drawing test her score was 7 and the score at Reisberg test was 4. Brain magnetic resonance imaging (MRI) showed a high signal on T2/ FLAIR sequences, restriction of water diffusion on DWI, low signal on ADC map at cortical level of right frontal-temporal-parietal-occipital and left superior frontal-parietal level (“cortical ribbon” sign) and right caudate nuclei (**Fig. 1**). 

**Fig. 2:**
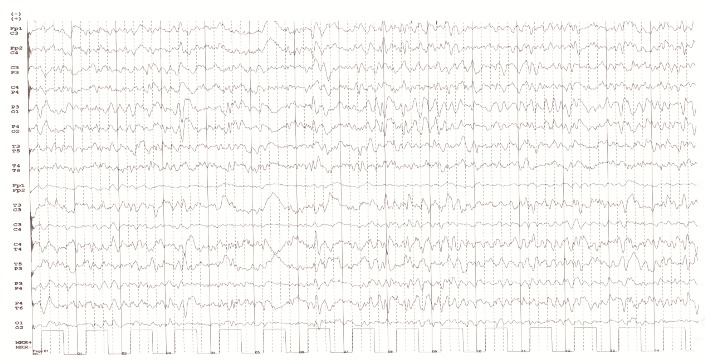
Repetitive sharp waves with a mean frequency of 1.0 Hz and a mean voltage of 120 μV

Biochemistry and cell blood count were normal. The analysis of the cerebrospinal fluid (CSF) showed 2 polymorphonuclear leukocytes/ mm3, normal glucose and slightly increased protein (0.5 g/ l, normal range 0.1-0.3 g/ l). In CSF, 14-3-3 protein was positive (ELISA), amyloid β1-42 was within the normal range (563 pg/ ml, normal range > 375 pg/ ml) and the level of tau protein was high (1580 pg/ ml, normal range <1300 pg/ ml). Serum antibodies against treponema pallidum and human immunodeficiency virus, Epstein Barr virus, cytomegalovirus were negative. Real time PCR from plasma and CSF for JC virus were negative, real time PCR from plasma was negative for cytomegalovirus, Epstein Barr virus, herpes simplex virus 1 and varicella zoster virus. Anti-neuronal antibodies and tumor markers were negative. Electroencephalography showed periodic sharp wave complexes (**Fig. 2**).

After one week, the patient presented visual and auditory hallucinations, motor automatisms, intermittent inattention, rapid cognitive deterioration, and relative change in the circadian rhythm, with increased daytime sleepiness. By the third week, she developed akinetic mutism with myoclonic jerks at nociceptive stimuli. The patient was taken home by her family and she died at short time after hospital release.

**Case 2**

**Fig. 3:**
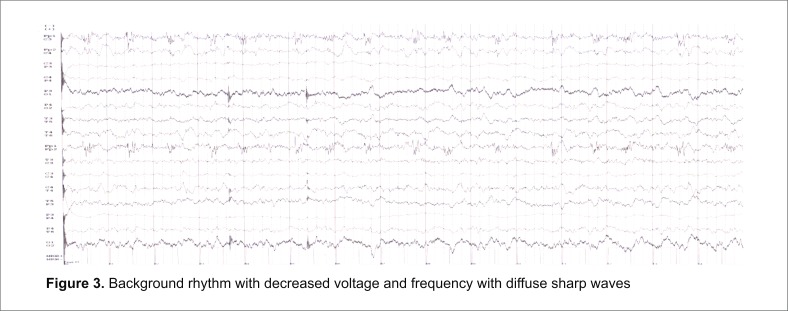
Background rhythm with decreased voltage and frequency with diffuse sharp waves

A 67-year-old woman had presented progressive onset of vertigo with unsteady gait, memory impairment, and emotional lability followed by right motor deficit and language disorder two months before admission. The medical history included arterial hypertension, psoriasis vulgaris and the family history was negative for dementia or insomnia. The patient had had insomnia for three years; she usually slept only two hours per night. Three weeks before admission, the patient consulted a neurologist from another hospital where she was diagnosed with depression and was treated with Escitalopram 50 mg once daily. The neurological examination revealed right visual neglect, right hemiparesis, slow initiation of movements, ataxia of right limbs, receptive aphasia (she executed simple commands but made errors at complex orders and presented intoxication with commands), dyscalculia and memory impairment for recent data. The neuropsychological assessment showed severe cognitive deficit, temporal and spatial disorientation, attention deficit, anterograde hypomnesia, dyscalculia, dysexecutive syndrome, dysgraphia, visuospatial disorganization. At MMSE, the patient’s score was 13 and 2 at Clock-drawing test, Reisberg score was 5. The routine laboratory tests were within normal range. Brain MRI revealed a lesion with high signal on DWI, T2, FLAIR sequences at the level of left putamen (**Fig. 1**). Cerebrospinal fluid examination showed normal cells count, glucose and protein, increased 14-3-3 protein (72729 AU/ ml on ELISA and positive on Western blot), low amyloid β1-42 (325 pg/ ml, normal range >450 pg/ ml) and elevated tau protein (1380 pg/ ml, normal range <1300 pg/ ml). The EEG showed initially background slowing, predominant frontal, followed by the appearance of PSWC five days later (**Fig. 3**). 

One week after admission, the patient presented myoclonus on the right side of the face, oculogyric seizures and, one week later, she became sleepy, responded to verbal stimuli but was unable to execute orders and presented an akinetic mutism. The patient was taken home by her family and died one day later. The histological examination of the brain tissue was refused by the patient’s family.

**Case 3**

**Fig. 4:**
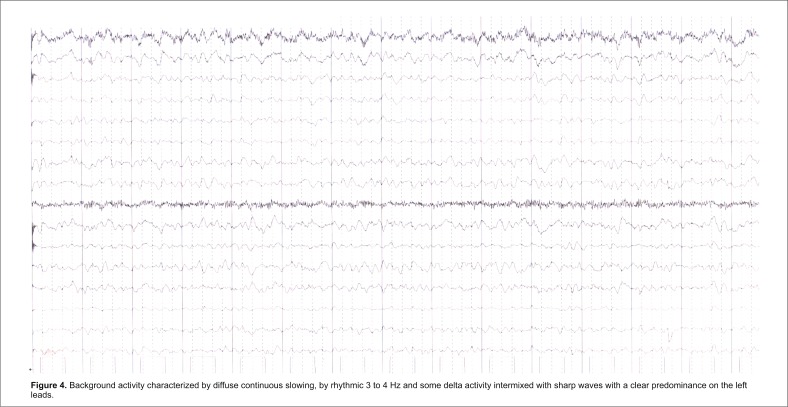
Background activity characterized by diffuse continuous slowing, by rhythmic 3 to 4 Hz and some delta activity intermixed with sharp waves with a clear predominance on the left leads

A 61-year-old woman was admitted for focal motor seizures and generalized convulsive seizures, slowly progressive cognitive decline, and psychomotor agitation. The patient was treated for depression and psychotic manifestations. Neurologic examination revealed confusion state, behavioral disorder, left hemiplegia, right hemiparesis (3/ 5 BMRC), focal motor seizures on the left side with version of the head and eyes towards the right side, bilateral pyramidal syndrome. Routine laboratory tests were normal. Brain MRI revealed water restriction on DWI sequences at the level of the left caudate nucleus and high signal on T2/ FLAIR bilaterally in frontal, parietal and temporal cortical areas (“cortical ribbon” sign) (**Fig. 1**). Cerebrospinal fluid examination showed 1 polymorphonuclear/ mm3, normal glucose and protein, high levels of 14-3-3 protein (60205 AU/ ml on ELISA and positive on Western blot), normal amyloid β1-42 (600 pg/ ml, normal range >450 pg/ ml) and increased tau protein (1560 pg/ ml, normal range <1300 pg/ ml). The EEG showed intermittent right lateralized frontotemporal PSWC followed, in eight days, by background flattening (**Fig. 4**). 

The patient continued to have seizures and, two weeks later, became sleepy, with dysphagia and global aphasia, akinetic mutism and died one week later. The family refused autopsy.

**Case 4**

**Fig. 5:**
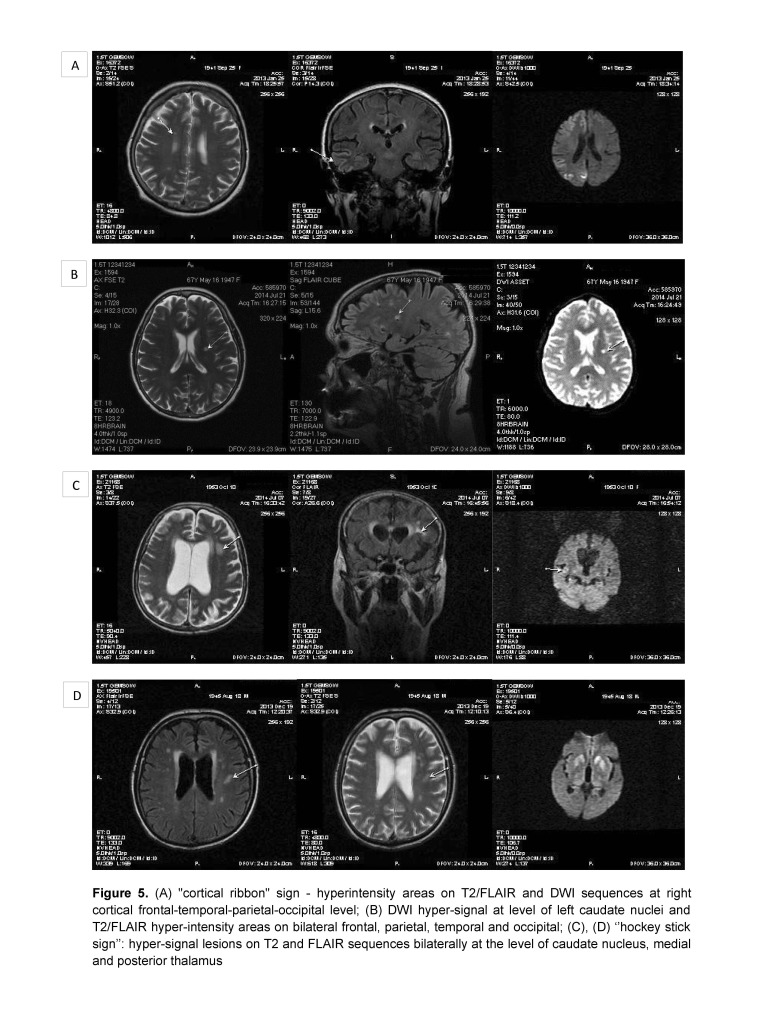
(A) “cortical ribbon” sign – hyperintensity areas on T2/ FLAIR and DWI sequences at right cortical frontal-temporal-parietal-occipital level; (B) DWI hyper-signal at level of left caudate nuclei and T2/ FLAIR hyper-intensity areas on bilateral frontal, parietal, temporal and occipital; (C), (D) “hockey stick sign”: hyper-signal lesions on T2 and FLAIR sequences bilaterally at the level of caudate nucleus, medial and posterior thalamus

A 68-years-old man presented a progressive onset, approximately three months before admission, of cognitive decline, postural instability, dysarthria, dysphonia, and diplopia. In the last three weeks, the patient had been unable to maintain an upright position, in the last two days he had became unable to speak, eat, and had presented a motor deficit of the four limbs. Two years before, the patient had been diagnosed with Parkinson’s disease and was treated with levodopa-benserazide, but without improvement. The medication was stopped because of a hallucinatory episode that had been remitted afterwards. The neurologic examination revealed a clinical picture of akinetic mutism and myoclonus. The brain MRI showed high signal lesions on T2, FLAIR and DWI sequences bilaterally at the level of caudate nuclei, medial and posterior thalamus (aspect of “hockey stick” sign, and cortical frontal parasagittal (**Fig. 1**). Routine laboratory tests were within normal range. Cerebrospinal fluid examination showed normal glucose, 632 mg/ dl albumin (normal range <350 mg/ dl), 2 polymorphonuclears/ mm3. Protein 14-3-3 was positive in CSF (ELISA), amyloid β1-42 was normal (550 pg/ ml, normal range>450 pg/ ml, and tau protein was elevated (1640 pg/ ml, normal range <1300 pg/ ml). Serum anti-neuronal antibodies profile and anti-glutamate receptor antibodies were negative. Electroencephalography showed slow waves on the left frontal, temporal and parietal leads on a background slowing of the rhythm and after two months the EEG showed a loss of alpha background, generalized slowing in the theta band and no PSWC (**Fig. 5A**). 

The patient died one week later. Autopsy was refused by the family. The clinical presentation with Parkinsonian syndrome generated the discussion about a possible association between two distinct pathologies characterized by abnormal protein deposition into the CNS. 

## Results and discussion

Our study included three females and one male, with a mean age of 67 years. The mean duration from the diagnosis until death was 3.2 weeks (range: 2-7weeks). After the exclusion of other pathologies, clinical evolution, EEG findings, MRI features and 14-3-3 protein dosage represented important criteria for establishing the diagnosis of probable sCJD. 

EEG findings

The EEG findings were variable among patients and during the evolution of the disease with background flattening or slowing in the alpha band, nonspecific abnormalities and appearance of PSWC especially in advanced phase in 3 out of 4 patients. The only patient who did not present PSWC on EEG had high signal lesions on basal ganglia and frontal “cortical ribbon” sign on DWI, T2, and FLAIR sequences. 

Brain MRI findings

The MRI showed cortical hyperintensities in at least two cortical regions on DWI, T2, and FLAIR in 2 patients, in one cortical region in 1 patient, and basal ganglia (putamen and/ or caudate) hyperintensities in all 4 patients (unilateral in 3 patients and bilateral basal ganglia lesions in 1 patient). Other studies [**9**,**10**] showed that the optimum diagnostic accuracy in the differential diagnosis of rapid progressive dementia was obtained when either at least two cortical regions (temporal, parietal or occipital) or both caudate nucleus and putamen displayed high signal in FLAIR and DWI sequences on MRI (positive in 83% of the cases). 

CSF biomarkers for CJD

In all four cases, the 14-3-3 protein was positive, amyloid β1-42 was normal and tau protein was increased. Other studies reported lower 14-3-3 specificity in discrimination of CJD from acute neurological events (82–87%), high 14-3-3 specificity in neurodegenerative diseases (95–97%) and non-neurological conditions (91–97%) [**7**]. The combination of the 14-3-3 protein with the tau protein is reported to significantly increase the specificity: high 14-3-3 protein levels associated with low tau levels in the CSF might be indicative of a potentially treatable inflammatory or autoimmune mediated disorder [**7**]. The elevated levels of tau and phosphorylated tau in CSF are recognized hallmarks for Alzheimer’s disease (AD) [**11**]. For unusual phenotypes of AD suggesting CJD, the determination of total CSF prion protein (t-PrP) levels increased the diagnostic accuracy [**12**].

Clinical symptoms and course of illness

The clinical features and duration of the disease varied in our cases. The clinical onset was with visuospatial disorientation, conduction aphasia, spatial neglect, insomnia, depression, seizures, behavioral disorder, Parkinsonian syndrome. During evolution, the other neurological signs such as the pyramidal syndrome, visual and oculomotor disorders, myoclonus, and akinetic mutism have appeared. 50% of the patients in our study have died in the first three weeks after diagnosis with a range between 2 to 7 weeks. Numerous papers reported various clinical presentations of CJD: Heidenhain’s syndrome with visual disorder speech disturbance and aphasia, cerebellar disturbance, pure psychiatric onset, stroke-like onset [**13**,**14**], movement disorders ranging from dystonia, chorea, parkinsonism, tremor to myoclonus [**15**], convulsive and nonconvulsive status epilepticus. 

Our study revealed that characteristic EEG findings (periodic sharp wave complexes) have non-uniform sensitivity across the clinical spectrum of sCJD and brain MRI, especially DWI, is a reliable diagnostic tool, but there is a need for new biomarkers for the clinical diagnosis of CJD.

**Acknowledgements**

Authors would like to thank Alina Stuparu, MD, who treated one of the presented patients, Ioana Mindruta, MD, who contributed to the interpretation of electroencephalography, Melania Dulamea for preparing the figures and tables.

**Conflict of interest**

None
